# Antimicrobial Peptides in Gastrointestinal Inflammation

**DOI:** 10.4061/2010/910283

**Published:** 2010-11-25

**Authors:** Simon Jäger, Eduard F. Stange, Jan Wehkamp

**Affiliations:** Department of Internal Medicine I, Robert Bosch Hospital, Dr. Margarete Fischer-Bosch-Institute of Clinical Pharmacology, Auerbachstr. 112, 70376 Stuttgart, Germany

## Abstract

Acute and chronic inflammations of mucosal surfaces are complex events in which the effector mechanisms of innate and adaptive immune systems interact with pathogenic and commensal bacteria. The role of constitutive and inducible antimicrobial peptides in intestinal inflammation has been investigated thoroughly over the recent years, and their involvement in various disease states is expanded ever more. Especially in the intestines, a critical balance between luminal bacteria and the antimicrobial peptides is essential, and a breakdown in barrier function by impaired production of defensins is already implicated in Crohn's disease. In this paper, we focus on the role of antimicrobial peptides in inflammatory processes along the gastrointestinal tract, while considering the resident and pathogenic flora encountered at the specific sites. The role of antimicrobial peptides in the primary events of inflammatory bowel diseases receives special attention.

## 1. Introduction

Although a host of different bacteria colonizes the gut from the oral cavity to the rectum, translocation of bacterial agents through the intestinal walls remains limited to highly pathogenic bacteria or predisposing disease states in which the natural defense mechanisms are compromised. 

In the healthy individual, the physical barrier created by the thin layer of epithelium forms the basis of the mucosal defense. In addition, the production of an array of antimicrobial peptides by secretory epithelial cells limits the invasion and adherence of pathogenic and commensal bacteria. Salient examples of antimicrobial peptides are the defensins and cathelicidin LL37, the two major classes of AMPs in mammals, yet other molecules like elafin or secretory leukocyte protease inhibitor (SLPI) complement the effector mechanisms of innate and adaptive immune systems. Equally important, in the small and large intestine, goblet cells are responsible for the production of highly glycosylated proteins, which form a gel-like layer over the surface epithelium. The outer portion of this layer is heavily colonized by bacteria, whereas the inner stratum's low bacterial load results from the high local levels of antimicrobial peptides [1].

Recent years have seen a steadily rising interest in antimicrobial peptides, and their implication in the pathogenesis of intestinal processes like Crohn's disease [2] or necrotizing enterocolitis [3] as well as their role in psoriasis and atopic dermatitis, cystic fibrosis and otitis media has garnered the attention of an increasing group of scientists. 

In this paper, we would like to focus on the role of antimicrobial peptides in inflammatory processes along the gastrointestinal tract, while considering the resident and pathogenic flora encountered at the specific sites. The role of antimicrobial peptides in the pathogenesis of the idiopathic inflammatory bowel diseases receives special attention. 

## 2. Antimicrobial Peptides of the Gastrointestinal mucosa

### 2.1. Defensins

Defensins serve as endogenous antibiotics with microbicidal activity against Gram-negative and Gram-positive bacteria, fungi, viruses, and protozoa [4]. One of their fundamental characteristics is the presence of three intramolecular disulfide bonds. The pattern of linkage between the cystein residues allows the classification into two major groups, the *α*-defensins and the *β*-defensins (the cyclic octadecapeptide called *θ*-defensin were not found in humans so far). The former are linked by a 1–6, 2–4, 3–5 pattern, the latter 1–5, 2–4, 3–6 pattern, yet their three-dimensional structure is similar [5, 6]. The total of six *α*-defensins includes human neutrophil peptides 1–4 (HNP1–4) produced by granulocytes and human defensin 5 and 6 (HD5 and HD6) produced by Paneth cells. It should be noted that the Paneth cell defensins are stored as propeptides and require cleavage by trypsin, which is stored in Paneth cell granules as a zymogen as well [7, 8]. 

*β*-defensins are mainly produced by epithelial cells [9], and four subtypes, designated hBD-1 to hBD-4, have been identified in the human mucosa so far. hBD-1 is ubiquitously expressed at all surfaces of the human body including the skin, the respiratory, urogenital and the gastrointestinal tract [10–15]. hBD-2 and hBD-3 are inducible antimicrobial peptides expressed by enterocytes throughout the intestinal tract on demand. 

Biochemical properties of the human defensin family include a low molecular mass from 3 to 6 kDa and a cationic charge, which allows these molecules to bind to negatively charged phospholipid groups on microbial surfaces. The exact mechanism by which defensins exert their bactericidal effect has still not been identified, but it has already become clear that they do not act with a uniform mechanism. One model, the “Shai-Matsuzaki-Huangh” model, proposes that after integration of defensins into the cell membrane, its outer layer expands and strains the inner leaflet of this bilayer, leading to disruption or formation of toroidal pores [16]. On the other hand, hBD-3 has been shown to function rather by inhibiting steps of the biosynthesis of the bacterial cell wall [17].

Defensins were also noted for their chemotactic properties. The chemoattractant effect on immature dendritic cells and CD4+ T cells has been shown to act through chemokine receptor CCR6 [18]. Chemoattraction of macrophages and monocytes has been observed as well, but these cells do not express CCR6. A recent publication now reported that hBD-2 and hBD-3 are chemotactic for these cell lines in a CCR2-dependent manner [19]. Other investigations have shown that hBD-2 induces the migration of mast cells by activating G-protein-phospholipase C-coupled receptors and is a specific chemoattractant for human neutrophils [20, 21].

In a broader concept, Peyrin-Biroulet and Chaimallard position defensins at the interface between innate and adaptive immunity, proposing that NOD2-mediated microbial recognition leads to secretion of defensins, which in turn attract immature dendritic cells, help in their maturation and promote the subsequent activation of T cells [22].

Also, HD-5 may influence the intestinal inflammatory response by binding to the cell membrane of intestinal epithelial cells. A subsequent induction of interleukin-(IL-) 8 was observed in a concentration- and structure-dependent fashion [23, 24].

### 2.2. Cathelicidins

The second major group of AMPs in mammals are the cathelicidins. While a signal peptide called “cathelin prosequence” can be found at their N-terminus, the C-terminal part is formed by a more variable cationic region that has antimicrobial activity once cleaved from the holoprotein. The only cathelicidin identified in humans was termed LL-37/h-CAP18. Its constitutive expression is found in various immune cells, in salivary glands, and in epithelia of respiratory, digestive and reproductive tracts while keratinocytes and intestinal cells can be induced to enhance expression. LL-37's antimicrobial properties are supplemented by its chemotactic effect on blood cells, activation of histamin release from mast cells, or induction of angiogenesis [25].

### 2.3. Other Antimicrobial Peptides

Antimicrobial activity has been noted in a multitude of other small molecules. For example, the chemokines CCL14 and CCL15 are constitutively expressed at high levels in human intestinal epithelium and display potent antibacterial effects [24]. CLL20/macrophage-inflammatory-protein-3*α* and an additional 17 chemokines function as antimicrobials as well [26]. Elafin and secretory leukocyte protease inhibitor (SLPI) also exhibit broad spectrum antimicrobial activity against Gram-positive and Gram-negative bacteria, selected fungi and viruses [5], though in their principal role, these antiproteases serve to maintain tissue integrity by antagonising aggressive serine proteases like human neutrophil elastase (HNE) [27]. Yet another epithelial antimicrobial peptide is bactericidal/permeability-increasing protein (BPI), which is involved in lipid-mediated killing and the attenuation of proinflammatory signalling by bacteria. Its sphere of action covers mostly Gram-negative bacteria [28, 29]. For a quick overview, Table 1 lists the abovementioned antimicrobials along with their properties.

## 3. Antimicrobials in Gastrointestinal Diseases

### 3.1. Esophagus

Microbial infections of the esophagus represent a rather uncommon event in healthy individuals. Nevertheless, the immunocompromised host quite frequently suffers from infections with *C*. *albicans*, CMV or HSV, while bacterial infections remain rare. 

**Figure 1:**
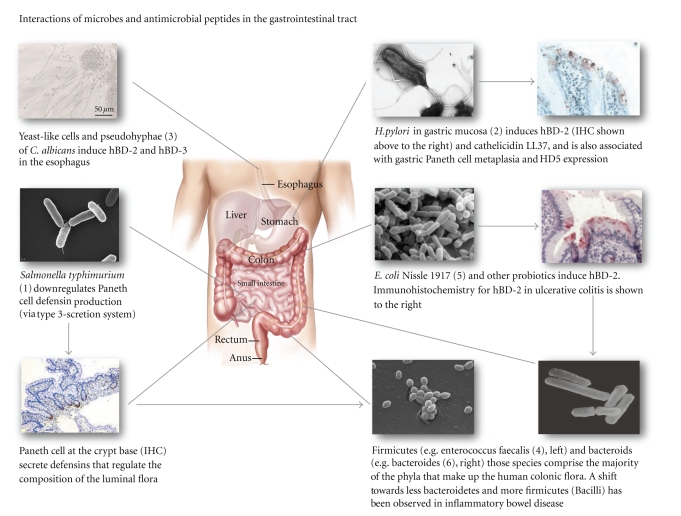


Fittingly, despite a high expression of numerous antimicrobial peptides, assays with oesophageal tissue showed a weakened potency to kill *C. albicans* [30], a fact which could help explain the susceptibility of esophageal tissues to infections with this yeast. Kiehne et al. [31] observed that *Candida* colonization induced a high expression of a subset of antimicrobial peptides, especially hBD-2 (shown in Figure 1) and hBD-3. In a subsequent mechanistic study the group showed that polymorphonuclear leukocytes (PMNs) reinforce the defensin expression in the epithelium. The authors speculate that individuals suffering from neutropenia lack this stimulus for the expression of epithelial antimicrobial peptides and thus, a pathophysiologic explanation for the high incidence of *Candida* esophagitis and *Candida*-related deaths in neutropenic patients can be proposed [32]. Furthermore, even in esophageal reflux disease, an induction of *β*-defensin expression (hBD-2 and hBD-3) could be found, although to a minor degree [31].

### 3.2. Stomach

The high prevalence and morbidity resulting from colonization by the Gram-negative bacterium *Helicobacter pylori* has captured much interest in the role of antimicrobial peptides in the stomach. Though the mucosa exhibits a strong inflammatory response against *H. pylori* bacteria, clearance of the pathogen is unsuccessful in many cases. 

*Helicobacter* infection is known to lead to a significant induction of hBD-2 (see Figure 1), while the defensin gene expression caused by non-*Helicobacter* gastritis is much less pronounced [33], a finding which was confirmed in a pediatric cohort [34]. In a recent study, it could be demonstrated that* H. pylori* induces gastric epithelial cells to upregulate the endogenous production of hBD-2 [35], furthermore the authors showed that this is mediated by the cytosolic pattern recognition receptor NOD1 (nucleotide-binding oligomerization domain 1). 

Also, an analysis of single nucleotide polymorphisms in the DEFB1 gene correlated patients with chronic active *H. pylori*-induced gastritis with the SNP G-52A, suggesting an involvement of the constitutive expressed hBD-1 in susceptibility to this form of gastritis [36]. In the setting of chronic *H. pylori* induced gastritis, intestinal metaplasia (replacement of the normal mucosa by a columnar epithelium with characteristics of intestinal epithelia, e.g., goblet cells, Paneth cells), is a frequent event. A high HD-5 expression has been observed by Shen et al. [37], suggesting that in intestinal metaplasia, where *α*-defensin producing Paneth cells are present, this metaplastic change may strengthen the antibacterial response via production of HD-5. Aside from the defensins, *H. pylori* is reported to induce Cathelicidin LL-37 in gastric epithelial cells [38].

### 3.3. Inflammation of the Biliary Tree

10%–20% of adult populations in developed countries suffer from cholelithiasis (gallstones). Though more than 80% of patients remain asymptomatic, infections of the gallbladder or the biliary tree are common diseases, which require antibiotic treatment in many cases. The normal sterility of bile is maintained by the bactericidal effect of bile salts and immunoglobulin A, and a notable expression of hBD-1 and hBD-2 is documented in biliary tract epithelium and in the liver [39]. Similarly to other anatomic sites, hBD-1 expression is constitutive, while in the large intrahepatic bile ducts, hBD-2 was induced by biliary obstruction or hepatolithiasis, where these peptides contribute to the local antimicrobial defense. 

Interestingly, in the epithelium of four of five patients with primary sclerosing cholangitis and in all controls with normal histology [39], hBD-2 expression remained low. Furthermore, in all bile samples which were analysed, hBD-1 could be found constitutively, while hBD-2 was confined to those with hepatolithiasis [39]. Patients with primary sclerosing cholangitis, especially following endoscopic manipulation, suffer from frequent bouts of infection. Although further studies are needed, the observed lack of induction of hBD-2 and possibly other antibacterial peptides could be implicated in the disease mechanism. 

D'Aldebert et al. found an intense immunostaining for cathelicidin in human liver biliary epithelium, and showed that bile salts (chenodeoxycholic acid and ursodeoxycholic acid), which also possess intrinsic bactericidal properties, induce cathelicidin expression through different nuclear receptors. According to their results, either farnesoid X receptor or vitamin D receptor is involved and upon activation, promote cathelicidin expression in the biliary tract [40].

### 3.4. Intestine

The microbial colonization of the lumen increases along the intestine, though the number of bacteria is still very low from the duodenum to the proximal ileum. The distal ileum contains up to 10^8^ primarily anaerobic bacteria per gram of luminal contents [41], whereas up to 10^11^–10^12^ bacteria per gram colonize the colon. The bacterial microflora is crucial for the maintenance of human health and the development of the mucosal immune system. Moreover, its contribution to the pathogenesis of the chronic idiopathic inflammatory bowel diseases is widely acknowledged. In these entities, a shift in microbial composition towards less Bacteroidetes and more Firmicutes (Bacilli) has already been observed (see Figure 1).

#### 3.4.1. Small Intestinal Inflammation

On the one hand, the scarcity of bacteria in the ileum can be attributed to the hostile environment created by acid, bile, and pancreatic secretions as well as to the phasic propulsive motility of this part of the gut [42]. On the other hand, adaptive and innate branches of the immune system contribute as well to maintain a low microbial density. Paneth cells, which are a characteristic epithelial lineage of the small intestine and localize to the bottom of the intestinal crypts, secrete *α*-defensins in response to bacterial antigens including lipopolysaccharide and muramyl dipeptide [43]. A constitutive expression of exceptionally high levels of *α*-defensins HD-5 and HD-6 could be demonstrated in human small intestines [44]. Interestingly, expression of HD-5 exceeds expression levels of other AMPs produced by the Paneth cell (lysozyme and sPLa2) by a factor up to 100 [45]. 

In studies with knockout animals, intestinal extracts from mice deficient for the cryptdin-processing enzyme matrilysin and thus lacking functional mature mouse *α*-defensins (the mouse homologs to defensins are called cryptdins), show decreased antimicrobial activity [46], and the authors furthermore observed that these mice are more susceptible to orally administered bacterial pathogens as well as to DSS-induced colitis. Other findings from a transgenic animal study revealed that human *α*-defensin HD-5 transgenic mice are resistant to infection from orally administered *S. typhimurium *[47]. Interesting in this context is the fact that *S. typhimurium* can downregulate HD-5 expression via a type-3 secretion system (see Figure 1).

In addition, the Paneth cell defensins can shape the composition of microbial species present in the small intestinal lumen, while the total number of bacteria remains unaffected [45, 48]. In a mouse model with transgenic expression of DEFA5, Salzman et al. demonstrated that the colonization with segmented filamentous bacteria (termed SFB, from the genus Clostridia) was dramatically decreased when the mice produced the human *α*-defensins HD-5. Interestingly in this context is the fact that mice colonized with SFB were shown to be more resistant to infection with *Citrobacter rodentium*, a close relative to the well-known *Escherichia coli*. Paneth cells also exert control over intestinal barrier penetration by commensals and pathogenic bacteria [49], apparently mediated by TLR (Toll-like receptor) recognition and a subsequent induction of antimicrobial peptides. The signalling was shown to be dependent on the expression of the MyD88 adaptor protein inside the Paneth cell. The release of Paneth cell secretions into the intestinal lumen thus follows stimulation of pattern recognition receptors (PRR, e.g., Toll-like receptors, NOD-like receptors, RIG-I-like receptors) with pathogen-associated molecular patterns, termed PAMPs, which are provided by resident and pathogenic bacteria. Corroborating the concept of a host driven composition of the microbial flora, Petnicki-Ocweija et al. showed that in the mouse model, the bactericidal activity of crypt secretions of the terminal ileum was severely compromised by NOD2 deletion, and that *NOD2* expression depends on the presence of commensal bacteria [50]. 

The human NOD2 protein (nucleotide-binding oligomerization domain/caspase recruitment domain (NOD/CARD) is a cytoplasmic receptor for bacterial molecules which is predominately expressed in Paneth cells [51]. NOD2 received great attention after it was identified as a susceptibility gene for Crohn's disease in 2001 [52, 53]. Structural changes in the leucine-rich repeat region of NOD2 result from two single nucleotide polymorphisms (SNPs) and an insertion mutation that leads to a frameshift mutation at Leu1007 (L1007fsinsC). The authors found that homozygosity or compound heterozygosity increases the relative risk for Crohn's disease by as much as 40-fold compared with individuals without mutation. Approximately one third of patients affected by ileal Crohn's disease show mutations in the NOD2 status [22]. Of note, the three common allelic variants of the NOD2 gene were correlated with an increased susceptibility only in Caucasians and studies have shown remarkable differences in the genetic variability of the NOD2 gene in different ethnical populations. The three common variations could not be found in Asian populations [54, 55] and in African Americans mutation frequency as well as the attributable risk were much lower [56]. These findings could partially explain variations in the frequency of Crohn's disease in different world populations. 

A link among Crohn's disease, NOD2, and *α*-defensins is strongly suggested by observations made in NOD2-knockout mice which exhibit a decrease in Paneth cell defensins (cryptdins) alongside an impaired mucosal immune response to orally delivered but not intraperitoneally administrated *L. monocytogenes* [57]. Also, a decreased *α*-defensin mRNA expression in biopsy specimens of ileal Crohn's patients, which was even more pronounced in patients carrying NOD2 mutations [58, 59], was observed. The decrease in *α*-defensins was independent of inflammation in the specimens and not observed in ulcerative colitis or pouchitis, an inflammatory control of non-Crohn's ileitis. Of note, patients with colonic Crohn's showed unchanged levels of *α*-defensins in biopsies from their ilea [60], Wehkamp 2005). 

In contrast to these results, Simms et al. dispute the notion that reduced *α*-defensin mRNA expression is a primary effect. The authors propose that this finding is due to epithelial loss, as they did not observe reduced *α*-defensin levels in noninflamed ileal mucosa in CD patients [61]. Furthermore, the association between NOD2 mutation (L1007fsinsC) and particularly low *α*-defensin levels could not be reproduced in their cohort. 

Yet, in an assessment of luminal HD-5 levels in ileostomy fluids, significantly lower defensin levels in Crohn's patients than in controls were observed, and especially in those with homozygous/compound heterozygous NOD2-mutations [62]. Elphick et al. reported furthermore that in Crohn's disease, the processing of pro-HD-5 to mature HD-5 by trypsin is impaired. HD-5 in the ileostomy fluid of Crohn's patients is predominantly present in complexes with trypsin or chymotrypsin, suggesting an additional way by which epithelial defense might be compromised in CD.

Other mechanisms leading to diminished Paneth cell *α*-defensin function in patients with ileal CD are even more complex. The differentiation of crypt stem cells into mature secretory cells is governed by the so-called Wnt pathway. Disruption of this signaling cascade leads to impaired Paneth cell differentiation, an event which manifests as a disordered localization of these cells within the crypts [63]. One of the Wnt signaling transcription factors, TCF-4, shows a reduced expression in patients with ileal CD, independent of the extent of inflammation in the biopsies [64]. Further investigations revealed that in the ileal subset of Crohn's disease, a SNP in the TCF-4 promotor region (res3814570) was significantly more frequent than in colonic Crohn's or in ulcerative colitis [65].

A genomewide association study from 2007 identified ATG16L1 as susceptibility locus for ileal Crohn's disease [66]. ATG16L1 protein is involved in autophagy, a process which is essentially responsible for the degradation of intracellular structures, but also mediates degradation of phagocytosed or invasive bacteria. Moreover, Cadwell et al. provided evidence that in ATG16L1 knockout mice, granule exocytosis is abnormal [67, 68], and recently it has been shown that recruitment of ATG16L1 to the site of bacterial entry in the plasma membrane is dependent on activation of NOD2 by bacteria ([69]). As the Paneth cell's foremost activity is the secretion of huge amounts of defensins, an attractive interrelation can be proposed for the decreased defensin functionality in Crohn's disease and the mutation in ATG16L1. 

Genomewide screening could also identify X-box binding protein 1 (XBP1) as a risk factor for Crohn's disease and ulcerative colitis [70]. XBP1 deletion in mouse intestinal epithelial cells leads to an increased susceptibility to DSS-induced colitis and even spontaneous enteritis occurred [70].

Furthermore, an association study from Australia implicates KCNN4, a calcium-mediated potassium channel, in ileal Crohn's disease. This channel is involved in the secretory mechanisms in Paneth cells, and mRNA levels are reduced with NOD2 mutations [71]. 

Aside from these numerous associations between Paneth cells and defensins in ileal inflammation of CD, the involvement of antimicrobial peptides in active celiac disease, an inflammatory disorder of the small intestine as well, has been investigated. Vordenbäumen et al. assessed a panel of *β*-Defensins (hBD-1 to 4) and *α*-defensins (HD5-6) in duodenal biopsies of pediatric celiac disease patients and found a decreased hBD-1 and hBD-4 expression, while the remainder of the antimicrobial peptides did not show differences to healthy controls [34]. Although this observation confirmed previous investigations, the pathophysiologic significance of this expression pattern has yet to be determined.

#### 3.4.2. Colon

The composition of the extensive colonic microflora has been characterized more thoroughly by sequencing of 16S ribosomal DNA of fecal contents. Among the approximately 400 different species harboured by the human colon, two phyla clearly dominate: anaerobic Gram-positive firmicutes (Clostridium, Bacillus, Lactobacillus) and anaerobic Gram-negative bacteroidetes (Bacteroides, Flavobacteria) [72]. A high interindividual diversity has been noted, though at any time, each individual carries a stable “fingerprint” pattern [73]. Considering that an epithelium of only a single cell layer separates the bowel from the microbe-laden lumen, this barrier is remarkably effective. In addition to secreted immunoglobulins provided by the adaptive immune system, the innate branch offers a wide variety of antimicrobial peptides.

The first defensin identified in the human large bowel was the *β*-defensin hBD-1, and in the noninflamed colon, it is the major *β*-defensin. A recent publication reported that the peroxisome proliferator-activated receptor (PPAR) gamma is playing a major role in the constitutive expression of hBD-1 [74] and confirmed an earlier finding of a reduction of hBD-1 expression in inflamed mucosa of IBD patients [75]. Strongly supporting an important role of hBD-1 in colonic IBD, Kocsis et al. have reported a genetic association of hBD-1 SNPs with colonic Crohn's disease in a Hungarian cohort [76]. These findings challenge the perspective that reduced defensin expression is merely the result of epithelial loss in inflammatory states [77].

In the healthy colon, hBD-2 and hBD-3 are absent and only induced during inflammation or infection. Stimuli for hBD-2 induction comprise both bacteria and cytokines, like *Campylobacter jejuni *[78] or the bacterial component flagellin from the E. coli strain Nissle 1917 (shown in Figure 1), which is used as probiotic in the maintenance treatment of ulcerative colitis [79]. On the cytokine level, the induction is mediated by proinflammatory cytokines such as IL-1*β* (through NF-*κ*B-dependent and AP-1-dependent pathways) and TNF-*α* [80] or IL-17 [81].

Different defensin mRNA expression in the different forms of inflammatory bowel diseases has been noted, as in patients with ulcerative colitis, hBD-2 and hBD-3 are strongly induced in the event of inflammation. In comparison, the induction is attenuated in Crohn's disease [14, 82, 83] and the colonic mucosa of Crohn's disease patients is compromised in the killing capacity towards different commensal bacteria [84]. The mechanism behind the reduced hBD-2 expression in inflamed colonic Crohn's has not been elucidated up to now. In a European and US cohort, gene copy numbers for hBD-2 [85] were reduced, but results from a New Zealand Cohort were inconsistent with this finding [86].

As NOD2 mutations have been generally associated with Crohn's disease, researchers also investigated the effects of NOD2 mutations on the expression of *β*-defensins. Voss et al. demonstrated that the expression of hBD-2 is mediated by NOD2 activation [87], but a subanalysis stratified for NOD2 mutation status could not identify differences in colonic hBD-2 expression (Wehkamp, unpublished observation). Further investigations revealed that 1,25-dihydroxyvitamin D3 and MDP induce expression of hBD-2 and cathelicidin through stimulation of NOD2 expression [88].

Many lines of evidence thus point to a major role of *β*-defensins in inflammatory processes of the colon. New data for the *α* *-defensins* from a mouse model show that the Paneth cell cryptdins synthesized in the ileum retain their structure and functionality till the colonic lumen [89], suggesting a role for *α*-defensins in the large bowel as well. As has been mentioned above, Paneth cell metaplasia is noted on different sites of inflammation along the gastrointestinal tract, including the colon [90]. This metaplastic response could therefore represent a mechanism that provides additional protection by *α*-defensins at these sites. Furthermore, an interesting observation by Langhorst et al. showed a significant elevation of hBD-2 peptide in fecal samples from patients with irritable bowel syndrome, a condition which demonstrates no macroscopic visible inflammation on colonoscopy [91]. 

The antimicrobial peptide elafin shares a similar expression pattern with the inducible *β*-defensin, LL37 and secreted leukocyte protease inhibitor. Its additional function as an antiprotease balances the proteolytic effects of HNE (human neutrophilic elastase) from polymorphonuclear cells in healthy tissues. Moreover, it has been found to be reduced in colonic Crohn's disease, which could point to an involvement of protease-antiprotease disbalance explaining in part the penetrating, transmural type of inflammation [92].

Cathelicidin (LL37) shows induction in inflamed tissues of ulcerative colitis, while in active Crohn's disease the induction seems to be attenuated [93]. In mutant mice, Cathelicidin restricts colonization with epithelial adherent bacterial pathogens like *Citrobacter rodentium* [94], confirming its vital role in the armamentarium of the innate immune system. 

The large intestine harbors a complex ecosystem, where classical immune cells and colonic epithelial cells interact in concert with the dense resident microflora [95]. After recognizing the importance of the microbiota in chronic intestinal inflammation too, the characterization of the enteric luminal flora in inflammatory bowel disease revealed differences in the composition compared to healthy controls. Swidsinski et al. [96] among others demonstrated that mucosa-associated bacteria are dramatically increased in IBD mucosa. Anaerobic *Bacteroides* species and aerobic *Enterobacteriaceae* (*E. coli*) were most prevalent and furthermore early disease recurrence seemed to be accompanied by increased numbers of *E. coli*, *Bacteroides,* and *Fusobacterium*.

## 4. Therapeutic Consequences

Taken together, defensins seem to be attractive targets for pharmacologic intervention in a range of diseases. In the case of inflammatory bowel disease, Kubler et al. examined the effect of the currently available treatments (immunomodulators like azathioprine, corticosteroids or aminosalicylates) on the expression of main antimicrobial defensins, but no significant changes could be observed [97]. Treatment with the anti-TNF antibody infliximab was reportedly associated with normalization of defensin mRNA expression [77], but this was interpreted as a general effect stemming from epithelial regeneration. New insights in the genetics of antimicrobial peptides and their respective pathways of induction, regulation, and secretion could lead to therapeutic strategies which aim to strengthen barrier defense on epithelial surfaces. As detailed above, in the Paneth cell, numerous mechanisms leading to defective *α*-defensin function have been identified and can offer sweet spots for directed therapies in the future. Probiotics are effective as a maintenance treatment in colonic IBD, which has already been shown in a placebo controlled, double-blind study with the bacterium *E. coli* Nissle 1917 [98]. A recent meta-analysis confirmed the beneficial effect of probiotic treatment in the maintenance of ulcerative colitis [99]. A possible mechanism is the induction of hBD-2, which has been demonstrated for *E. coli* Nissle, as well as for other therapeutic probiotic *E. coli* strains and *Lactobacilli* [100–102].

Moreover, induction of antimicrobial peptides by agents like worm eggs, vitamin D, specific bacteria, food, artificial components, or possibly prebiotics may also be helpful. A larger, dose finding phase II clinical trial with live ova from *Trichuris suis *(porcine whipworm) is about to be initiated in autumn 2010, as previous small studies have been shown to improve the clinical outcome in ulcerative colitis (a double-blind clinical study) and in Crohn's disease (an open-label study) [103, 104]. Iatrogenic infection with these parasitic worms (Helminths), which are not able to survive in the human intestine for longer than 12 days, is thought to modulate the immune response. Evidence for these findings come from the observation that children with helmintic infections have reduced atopy [105], and peripheral blood mononuclear cells increase the production of anti-inflammatory mediators IL-10 and TGF-*β* [106]. Whether the therapeutic effect is mediated by a shift in adaptive immune function, or whether stimulation of the production of antimicrobials is significantly involved, is an intriguing question. We hope to address this within the context of the aforementioned study. However, recent animal studies have provided evidence that infection with *Hymenolepis diminuta*, also known as rat tapeworm, can cause a significant disease exacerbation as well [107]. Thus, the therapeutic use of helminths in IBD has to be considered carefully and its risk potential has to be assessed meticulously. 

In any case new treatment alternatives for inflammatory bowel diseases are eagerly anticipated by patients and physicians alike, and most probably, advances will come from the field of innate immunity.

## Figures and Tables

**Table 1:** Antimicrobials in the gastrointestinal tract.

Antimicrobial peptide	Chromosomal location	Molecular mass (kDa)	Secretory stimuli	Distribution in gastrointestinal tract	Biological function	Changes in inflammatory bowel disease
hBD-1	8p23.1	3.5–4.5	Constitutive in epithelial cells, IFN-*γ* and LPS in monocytes	Ubiquitous in epithelial cells of small and large intestine, monocytes, monocyte-derived dendritic cells	Antimicrobial, chemotactic	Reduction in colonic IBD

hBD-2, 3, 4	8p23.1	3.5–4.5	LPS, flagellin mediated by NF-*κ*B and AP-1	Epithelial cells, monocytes	- Antimicrobial, chemoattractant for macrophages and monocytes, - hBD-2: mast cells and neutrophils	- Attenuated induction observed in colonic CD- Reduced copy numbers for hBD-2 in colonic CD

HD-5 and HD-6	8p23.1	3.5–4.5	NOD2 activation (MDP, LPS) TLR	Granules of ileal Paneth cells (also metaplastic Paneth cells in other areas of intestinal tract)	Antimicrobial, induction of IL-8	- Reduction in ileal CD, more pronounced in patients with NOD2 mutation- HD-5 and HD-6 expression due to metaplastic Paneth cells in UC and CD colon

Cathelicidin (“LL-37”)	3p21.3	18	Butyrate, vitamin D, bile acids, MDP	Epithelial cells, leukocytes	Antimicrobial, chemotactic	- Attenuated induction in colonic CD- Ileal CD and UC show regular induction

Elafin	20q13.12	9.8	IL-1, TNF-*α*	Epithelial cells, leukocytes	Antiprotease with antimicrobial and chemotactic properties	Attenuated induction in colonic CD

Secretory phospholipase A2	16p13.1–p12	14	LPS	Epithelial and inflammatory cells, Paneth cell granules	- Acute phase protein involved in eicosanoide metabolism- Small intestinal mucosal defense	?

Lysozyme	12q15	16.5	?	Gastric, pyloric and duodenal glands, small intestine, macrophages and monocytes, not in colonic tissue	Antimicrobial against Gram-positive bacteria, chemotactic	- Small intestine: no changes observed - Increased colonic expression due to metaplastic Paneth cells

BPI (bactericidal/permeability-increasing protein)	20q11.23	50	LPS	Epithelial cells, neutrophils	Antimicrobial, binds LPS-compounds	No changes observed, regular induction in IBD
